# Unlocking the Potential of *Pseudomonas aeruginosa* SWUC02: Cell-Free Supernatant and Extracts for Controlling Anthracnose Disease and Inducing Resistance in Yellow Chilli Seedling

**DOI:** 10.21315/tlsr2025.36.1.2

**Published:** 2025-03-30

**Authors:** Natthida Sudyoung, Papattananpak Thiratanabordeechot, Siritron Samosorn, Kulvadee Dolsophon, Onanong Pringsulaka, Supaart Sirikantaramas, Akira Oikawa, Siriruk Sarawaneeyaruk

**Affiliations:** 1 Department of Microbiology, Faculty of Science, Srinakharinwirot University, Watthana, Bangkok 10110, Thailand; 2 Department of Chemistry and Center of Excellence for Innovation in Chemistry, Faculty of Science, Srinakharinwirot University, Watthana, Bangkok 10110, Thailand; 3 Center of Excellence in Molecular Crop, Department of Biochemistry, Faculty of Science, Chulalongkorn University, Bangkok 10330, Thailand; 4 Graduate School of Agriculture, Kyoto University, Kyoto 606–8502, Japan

**Keywords:** Antifungal Activity, Chilli Anthracnose, *Colletotrichum truncatum*, Elicitor, Induced Systemic Resistance, Phenylalanine Ammonia Lyase

## Abstract

Chilli anthracnose is a significant constraint in chilli production and quality in cultivated areas worldwide. Alternative methods are being explored to avoid the use of chemical fungicides, including plant elicitors produced by beneficial microorganisms to enhance plant defense responses. However, studies on the use of biotic elicitors to control chilli anthracnose are limited. In this study, we investigated the efficacy of cell-free supernatant derived from *Pseudomonas aeruginosa* SWUC02 (CF-SWUC02) and its extracts on the antimicrobial activity and systemic resistance in yellow chilli seedlings. The anthracnose pathogen, *Colletotrichum truncatum* CFPL01, was isolated and assessed for its pathogenicity in yellow chilli and other varieties, exhibiting varying levels of susceptibility to anthracnose. CF-SWUC02 exhibited potential antimicrobial activity against several phytopathogens. Furthermore, it affected the mycelial growth and conidial germination of virulent *C. truncatum* CFPL01. The dichloromethane extract exhibited the highest efficacy in suppressing the growth of *C. truncatum* CFPL01, while the ethyl acetate extract demonstrated a significant reduction in anthracnose severity on both leaves and seedlings. The ethyl acetate extract also increased phenylalanine ammonia-lyase activity in treated seedlings, demonstrating the induction of plant immunity. In summary, the elicitor compounds present in CF-SWUC02 have the potential to reduce anthracnose severity either directly through pathogen inhibition or indirectly via stimulation of the plant defense responses. These findings provide valuable insights for the development of sustainable and effective strategies for the control of chilli anthracnose.

**Highlights**

- *Colletotrichum truncatum* CFPL01, a chilli anthracnose pathogen, was effectively suppressed by the cell-free supernatant of *Pseudomonas aeruginosa* SWUC02 (CF-SWUC02).
- The dichloromethane extract from CF-SWUC02 demonstrated strong potential against CFPL01.
- The ethyl acetate extract from CF-SWUC02 significantly reduced anthracnose symptoms and enhanced plant immunity by increasing phenylalanine ammonia-lyase activity in seedlings.

## INTRODUCTION

Chillies (*Capsicum* spp.) hold significant economic importance as a vegetable in Thailand owing to high domestic and international demand. Among Thai cuisine’s popular chilli varieties, yellow chilli (*Capsicum annuum* L.) stands out for its unique colour and flavour. In Thailand, chilli yield and quality are significantly affected by anthracnose disease, primarily caused by *Colletotrichum truncatum* (syn. *Colletotrichum capsici*), resulting in yield losses of up to 80% (Montri *et al*. 2009). *C. truncatum* is a hemibiotrophic pathogen that initially develops a short biotrophic phase followed by a necrotrophic phase, resulting in sunken areas, necrotic lesions and significantly decreased the fruit value. Severely infected plants may ultimately succumb to the disease (Saxena *et al*. 2016). Fungicides and integrated pest management strategies are commonly used to overcome epidemics. However, the excessive use of carbendazim and mancozeb for chilli anthracnose management has become a matter of concern due to the potential hazards they pose to human health and the environment, along with the propensity to promote the development of pathogen resistance. In comparison, biological approaches offer promising alternatives as they directly inhibit pathogens and possess the potential to enhance plant resistance. Additionally, the use of plant elicitors as part of biological control strategies can effectively stimulate plant defense mechanisms, further enhancing their ability to withstand pathogenic attacks.

Elicitors are substances that induce plant defense responses, primarily by promoting the accumulation of phytoalexins (Anand *et al*. 2009). The use of elicitors has become increasingly popular in sustainable agriculture because of their potential to reduce plant damage caused by phytopathogens and decrease the application of chemical pesticides. In plant defense systems, constitutive and long-lasting induced resistance mechanisms, such as systemic acquired resistance (SAR) and induced systemic resistance (ISR), can occur when plants are pre-treated with elicitors, resulting in resistance to subsequent pathogen attacks (Kamle *et al*. 2020). Currently, salicylic acid (SA) and its analogs have been recognised as plant elicitors that directly or indirectly enhance plant defense against pathogens when applied (Tripathi *et al*. 2019). Alternatively, elicitors of biotic origin, such as microbial secondary metabolites and cell-free cultures, can be used as biocontrol agents for plant diseases. An organocopper antimicrobial compound (OAC) and 3-hydroxy-5-methoxy benzene methanol (HMB), derived from *Pseudomonas aeruginosa*, can upregulate phenylalanine ammonia-lyase (PAL) expression and enhance plant resistance (dos Santos *et al*. 2021; Fatima & Anjum 2017). PAL is a key enzyme in the phenylpropanoid pathway, essential for synthesising various plant secondary metabolites that lead to broad-spectrum disease resistance in plants (Anand *et al*. 2009).

In our previous studies, we demonstrated the inhibitory effects of the plant growth-promoting bacterium *P. aeruginosa* SWUC02 and its cell-free supernatant (CF-SWUC02) on citrus canker pathogens. CF-SWUC02 effectively controlled canker disease development and viable bacterial cells activated plant defense-related genes (Sudyoung *et al*. 2020a; 2020b). *P. aeruginosa* SWUC02 genome contains various gene clusters involved in antimicrobial compound production. Moreover, it produces β-1,3 glucanase, which may inhibit fungal pathogens (Srisangchun *et al*. 2023a; 2023b). Despite *P. aeruginosa* being opportunistic, the antimicrobial activity displayed by CF-SWUC02 exhibited considerable potential. Expanding on these findings, this study aims to investigate the antimicrobial activity and resistance induction potential of CF-SWUC02 against anthracnose in yellow chilli caused by *C. truncatum*.

## MATERIALS AND METHODS

### Isolation and Identification of Fungal Pathogens

Anthracnose lesions on chilli (*C. annuum*) fruits were surface-sterilised with 0.6% v/v NaOCl, washed, and placed on 1.5% w/v water agar. Growing hyphal tips were then transferred to potato dextrose agar (PDA) amended with 200 μg/mL chloramphenicol. Colony morphology of pure isolates was observed on PDA. Isolates producing falcate-shaped conidia were subjected to the slide culture technique to examine acervuli, setae and appressoria. For fungal identification, genomic DNA was extracted using the cetyltrimethylammonium bromide (CTAB) method, followed by polymerase chain reaction (PCR) amplification of the internal transcribed spacer (ITS) region, partial β-tubulin (*TUB2*) and glyceraldehyde-3-phosphate dehydrogenase (*GAPDH*) genes with specific primer pairs (White *et al*. 1990; Woudenberg *et al*. 2009; Guerber *et al*. 2003). The obtained sequences were analysed using the Basic Local Alignment Search Tool (BLAST) against the NCBI GenBank database, and a phylogenetic tree was constructed using the maximum likelihood method with 1,000 bootstraps in MEGA X software version 10.2.6.

### Pathogenicity Assay on Yellow Chilli Variety

Yellow chilli fruits (approximately 10 cm in length) were surface-sterilised and inoculated with approximately 5,000 conidia using the pin-prick method (Montri *et al*. 2009). The inoculated fruits were covered with plastic bags and incubated at 25°C for 24 h with a 12-h photoperiod. Six days post-inoculation (dpi), the lesion area was measured using ImageJ software (version 1.53k) (Schneider *et al*. 2012). In the detached leaf assay, mature leaves were surface-sterilised and placed on water agar. The leaves were pricked, inoculated with fungal conidia and incubated at 25°C for four days. Yellow chilli seedlings were sprayed with fungal conidia (10^6^ conidia/mL), covered with plastic bags, and incubated at 25°C with a 12-h photoperiod. A 0.05% v/v sterile Tween 80 solution was used as a negative control. Disease incidence (DI%) was calculated as the percentage of infected samples among the total inoculated samples. The disease severity index (DSI%) was calculated using severity scores (Table 1) and the formula as described by Yang *et al*. (2009).

### Host Range Assessment on Other Chilli Varieties

A pathogenicity assay for CFPL01 was conducted on five chilli varieties popular in Thailand: Kheenoo Suan, red Jinda, Yuak, red Chee Fah and Num. All varieties belonged to *C. annuum*, except for Kheenoo Suan, which belonged to *C. frutescens*. Thirty fruits of each cultivar were infected with the pathogen. Surface-sterilised fruits were inoculated with fungal conidia, and the lesion size in the inoculated area was measured using ImageJ software. The DI% and DSI% were calculated.

### Preparation of CF-SWUC02 from *P. aeruginosa* SWUC02 Culture

The method of CF-SWUC02 production was carried out as described by Sudyoung *et al*. (2020a) with slight modifications. Briefly, the bacteria were grown in tryptone soya broth (TSB) supplemented with 0.01% w/v CuCl_2_ for six days at 30°C with agitation at 150 rpm. Subsequently, the culture was centrifuged to collect CF-SWUC02, which was stored at −20°C.

### Preliminary Screening of Antimicrobial Assay Against Phytopathogens

The antifungal activity of CF-SWUC02 against the isolated fungi was evaluated using the poisoned food technique. Eight fungal isolates were tested: *Cladosporium* sp. (YHPM01-1), *Alternaria* sp. (YHPM02-2 and YHPM03-1), *Fusarium* sp. (YHPB04-1 and YHPB05-1), *Stemphylium* sp. (YHPM06) and *C. truncatum* (CFPL01 and CFPL02). CF-SWUC02 was mixed with double-strength (2×) PDA in 6-well plates at 50% and 25% final concentrations. A mycelial plug was placed at the centre of each well. The plates were incubated at 28°C for four days. Mixed TSB supplemented with 0.01% w/v CuCl_2_ and 2x PDA served as a negative control. The fungal colony diameter was measured, and mycelial growth inhibition (MGI%) was calculated (Toghueo *et al*., 2016). The antibacterial activity of CF-SWUC02 was determined using the agar well diffusion method on tryptone soya agar (TSA) medium against *Agrobacterium tumefaciens*, *Pectobacterium carotovora*, *Ralstonia solanacearum* and *Xanthomonas citri* subsp. *citri* (*Xcc*). After 24 h incubation, the inhibition zone was calculated (Sudyoung *et al*. 2020a).

### Effect of CF-SWUC02 on Mycelial Growth and Conidial Germination of *C. truncatum* CFPL01

The antifungal efficacy was evaluated at various concentrations ranging from 50% to 0%, using a two-fold dilution approach. The fungal colony diameter was measured after four days of incubation using the poisoned food technique, and the MGI% was calculated. The effect of CF-SWUC02 on mycelial morphology was observed using the poisoned food technique in potato dextrose broth (PDB). CFPL01 was initially grown for two days and then inoculated in PDB with 50% CF-SWUC02. After three days, morphological changes were examined under a light microscope (400×). For conidial germination evaluation, 100 μL of fungal conidia were added to each well in 96-well plates containing 100 μL of CF-SWUC02 at concentrations ranging from 50% to 0%. After incubation at 20°C for 20 h in the dark, the germination rate was determined by examining 100 conidia, and the conidial germination inhibition (CGI%) was calculated using the formula described by Toghueo *et al*. (2016).

### Antifungal Activity of CF-SWUC02 Extracts and Determination of Minimum Inhibitory Concentration and Minimum Fungicidal Concentration

CF-SWUC02 was sequentially extracted twice with triple volumes of *n*-hexane, dichloromethane and ethyl acetate, followed by evaporation using a rotary evaporator. Antifungal activity against CFPL01 was tested at a final concentration of 200 μg/mL for each extract dissolved in 1% v/v dimethyl sulfoxide (DMSO). The minimum inhibitory concentration (MIC) and minimum fungicidal concentration (MFC) values of the culture extracts (ranging from 1,000 μg/mL to 1 μg/mL) were determined using the broth microdilution method in 96-well plates. Commercial fungicides, including benomyl 50 WP, carbendazim 50 WP and mancozeb 80 WP, were used for comparison. The MIC was defined as the lowest concentration without visible conidia growth after incubation for 48 h at 25°C. The MFC was evaluated by spotting 5 μL of the fungal suspension at the MIC and two concentrations above the MIC on PDA medium. The MFC was defined as the lowest concentration that completely eliminated fungal growth on fresh PDA medium after incubation for 72 h at 28°C.

### Potential of the CF-SWUC02 Extracts for Controlling Anthracnose Disease

In the detached leaf assay, excised leaves were surface-sterilised and pricked with a sterile needle. Subsequently, 10 μL of extract solution (200 μg/mL) was applied to the pricked areas. After 48 h of incubation, a conidial suspension of CFPL01 was applied to the treated areas. In the seedling assay, yellow chilli seeds were surface-sterilised and grown in a sterile mixture of vermiculate and peat moss (1:1 ratio) in plastic pots. The seedlings were incubated under ambient conditions, with a temperature of 25 ± 2°C, a dark:light cycle of 12:12, and a light intensity of 1,280 lx. After one month, the seedlings were sprayed with the extracts, followed by spraying with the fungal conidia 48 h after the extract treatment. The negative (mock) and pathogen (Col) groups were initially treated with a 2% v/v DMSO, followed by subsequent treatments of either 0.05% v/v Tween 80 or a conidial suspension, respectively. As part of the elicitor control, the initial treatment involved applying a 200 μM solution of SA. The severity scores were assessed (Table 1), and the DSI% was calculated.

### Phenylalanine Ammonia Lyase Assay

Seedlings grown under sterile conditions were treated with each extract, SA, or DMSO solution and incubated under a 12-h photoperiod at 25 ± 2°C for 48 h. Leaves were homogenised in liquid nitrogen. Subsequently, 0.5M Tris-HCl buffer (pH 8.8) containing 15 mM β-mercaptoethanol was added, followed by centrifugation at 15,000 × *g* for 10 min. The supernatant was used as the crude enzyme extract. The total protein was determined by the modified Lowry method (Waterborg 2009). To measure PAL activity, the protocol was slightly modified from Padró *et al*. (2021). The reaction mixture consisted of 0.5 M Tris-HCl (pH 8.8), 10 mM L-phenylalanine and the extract containing approximately 200 μg of protein. The absorbance at 290 nm was recorded at 0 min (pre-incubation) and at 60 min after incubation at 37°C, after which the reaction was terminated by adding 5 M HCl (post-incubation). The results were expressed as μmol mg^−1^ protein min^−1^.

### Statistical Analysis

Each experiment was conducted in triplicates. For parametric data, a one-way ANOVA followed by the LSD post-hoc test was employed at a 95% confidence level. On the other hand, for non-parametric data, the Kruskal-Wallis test was used, followed by the Mann-Whitney U test.

## RESULTS

### Pathogen Isolation and Identification

**Figure 1:**
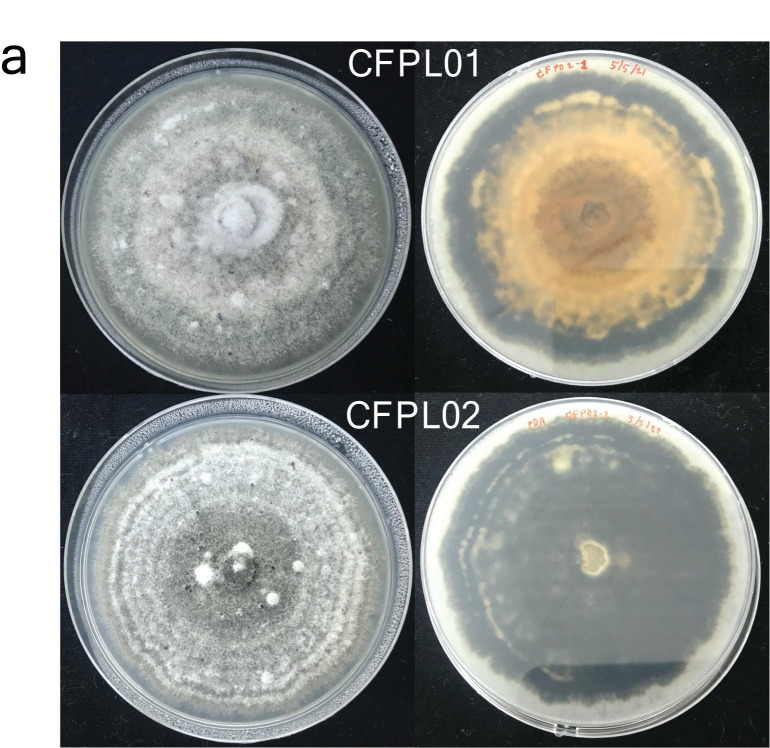

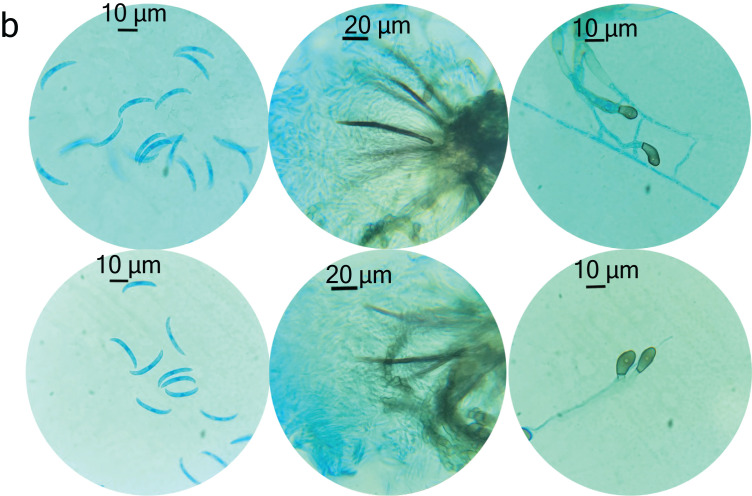

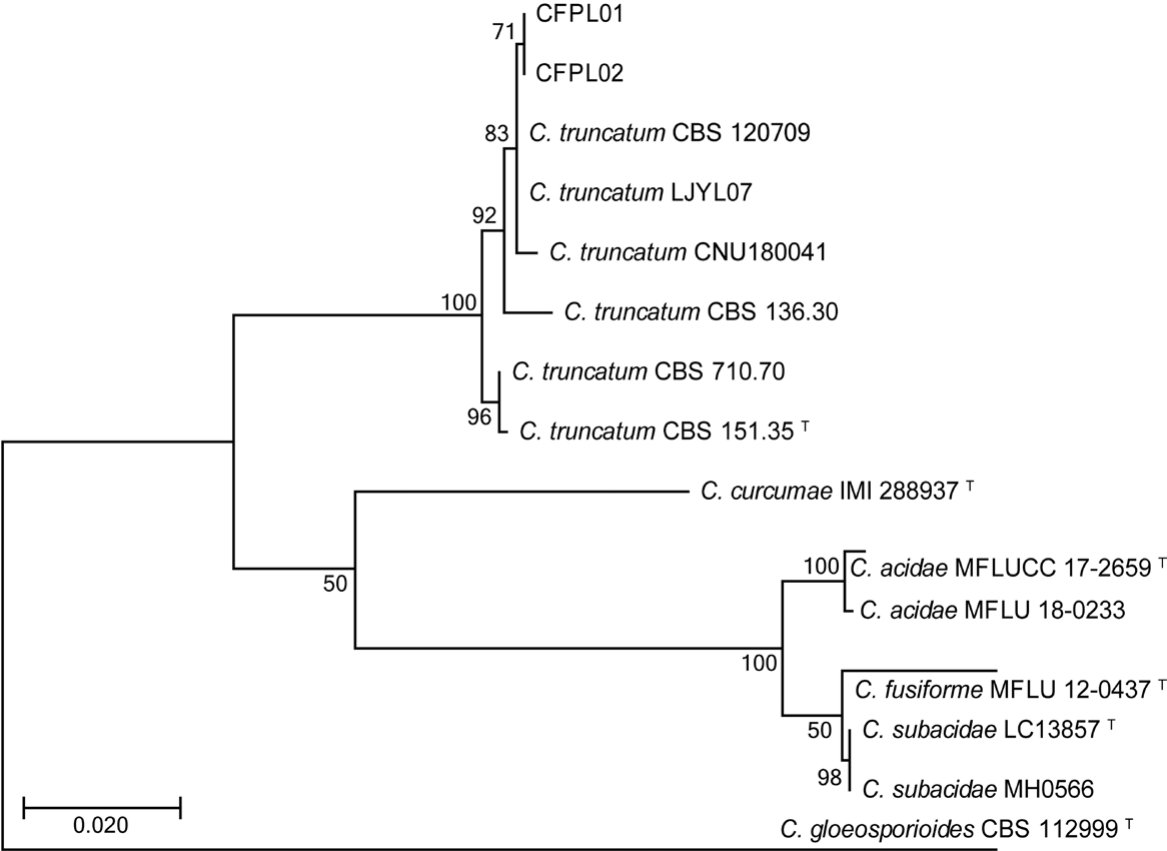
Identification of the fungal isolates. (a) Macroscopic characteristics of *C. truncatum* CFPL01 (upper) and CFPL02 (lower). Colony morphology on PDA captured from obverse (left) and reverse (right) sides; (b) Microscopic characteristics of *C. truncatum* CFPL01 (upper) and CFPL02 (lower). Conidia (left), setae and acervuli (middle) and appressoria (right); (c) Phylogenetic tree of the concatenated ITS, *TUB2* and *GAPDH* alignment, generated using the maximum likelihood method with the Tamura-Nei model and *C. gloeosporioides* as an outgroup species. The bootstraps consist of 1,000 replicates.

Eight fungal isolates were obtained from anthracnose-infected fruits. Only CFPL01 and CFPL02 were identified as *C. truncatum* based on their morphology and ITS, *TUB2* and *GAPDH* sequences. CFPL01 colony was grayish to dark gray with fluffy aerial mycelia on the obverse side, and pale orange to black on the reverse side, while the CFPL02 colony was light pale to dark gray with slight aerial mycelia on the obverse side, and black on the reverse side (Fig. 1a). Both produced one-celled, falcate-shaped, hyaline conidia measuring 18.7–27.5 μm × 2.2–2.8 μm (*n* = 30), and dark brown setae and acervuli. The appressoria were brown and elliptical to clavate in shape; some irregular (Fig. 1b). The phylogenetic tree confirmed the species (Fig. 1c). Additionally, six other isolates resembling *Colletotrichum* in colony morphology and/or conidial shape were identified as *Cladosporium* sp. (YHPM01-1), *Alternaria* sp. (YHPM02-2 and YHPM03-1), *Fusarium* sp. (YHPB04-1 and YHPB05-1) and *Stemphylium* sp. (YHPM06), based on ITS sequences. The ITS regions have been deposited in GenBank under the accession numbers OQ996545 to OQ996552. For *GAPDH* and *TUB2*, the accession numbers are OR018988 to OR018991.

### Pathogenicity Assay Performed on Yellow Chilli Fruits, Detached Leaves and Seedlings

**Figure 2:**
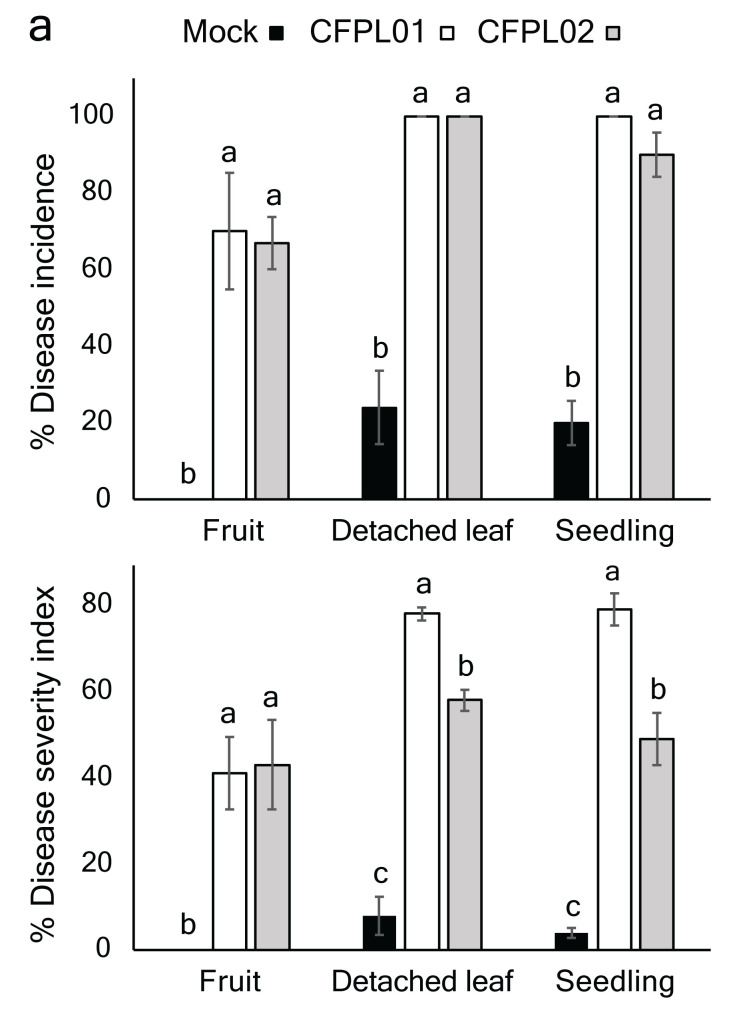

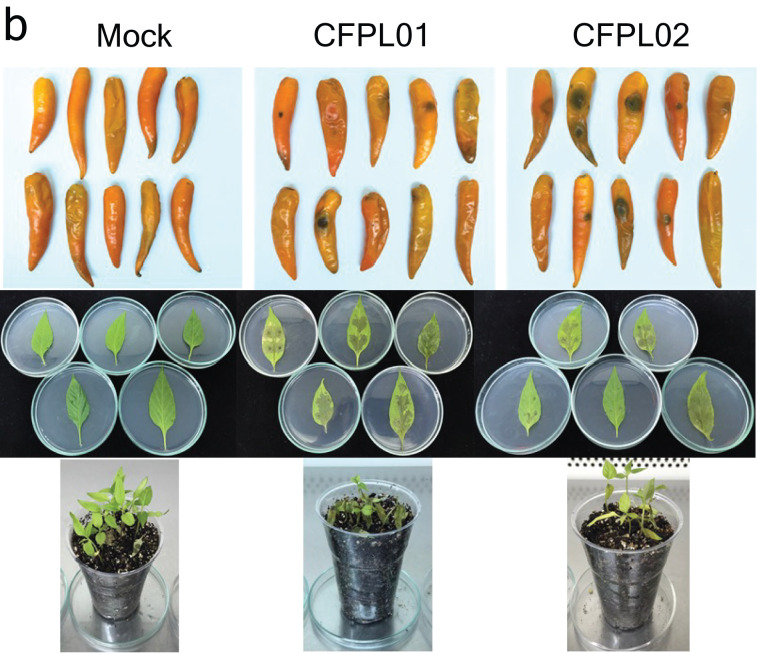

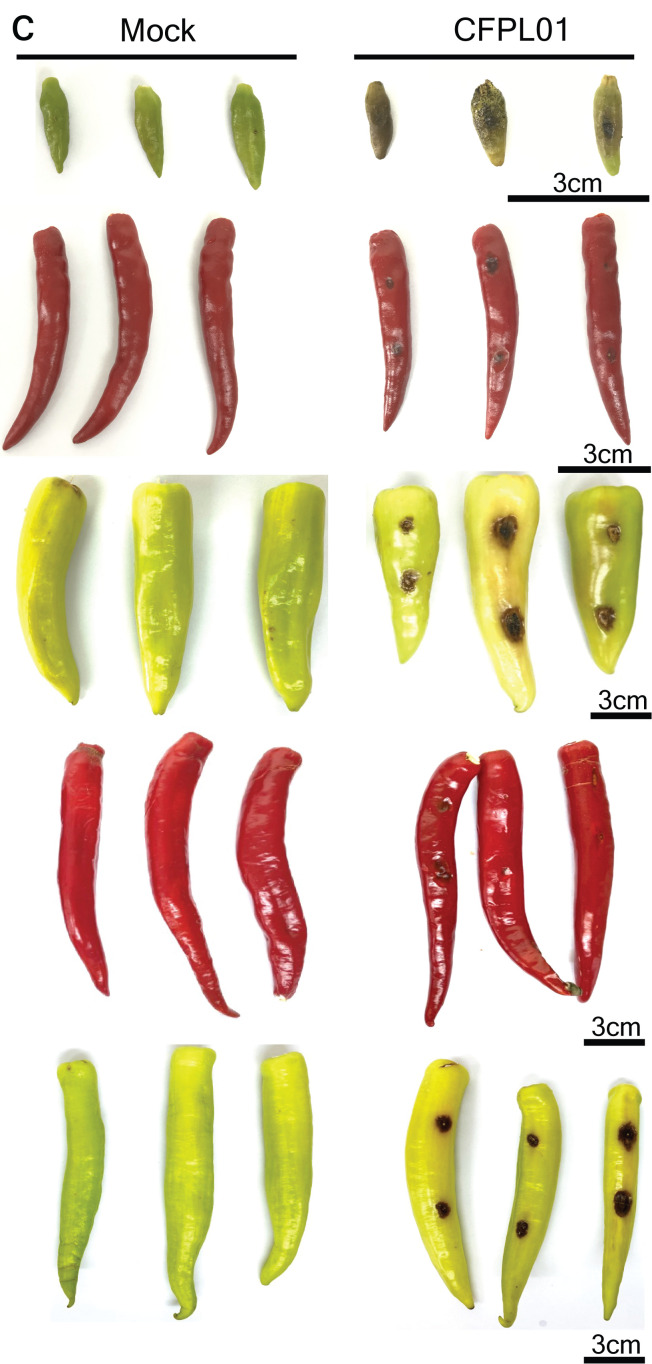
Pathogenicity of *C. truncatum* CFPL01 and CFPL02. (a) Percentage of disease incidence (upper) and severity index (lower) in yellow chilli. The bars represent the means with standard deviation. Different letters are used to denote significant differences among the treatments within each assay, as determined by Mann-Whitney U test (*p* < 0.05); (b) Disease symptoms on fruits (upper), detached leaves (middle), and seedlings (lower). Mock refers to the negative group; (c) Pathogenicity of *C. truncatum* CFPL01 on other chilli varieties. From upper, Kheenoo Suan, red Jinda, Yuak, red Chee Fah and Num, respectively. The left and right columns in each panel represent mock (negative group) and *C. truncatum* CFPL01-infected fruits, respectively.

Both CFPL01 and CFPL02 caused anthracnose symptoms in fruits, detached leaves and seedlings. The DI% values were similar and ranged from 67%–70% for fruits, 100% for detached leaves and 90%–100% for seedlings (Fig. 2a). In addition, CFPL01 exhibited higher DSI% value for detached leaves (80%) and seedlings (79%). In contrast, no significant difference was observed between CFPL01 (41%) and CFPL02 (43%) in the fruit assay (Figs. 2a and 2b). Hence, the CFPL01 was selected for further experiments.

### Host Range Assessment of *C. truncatum* CFPL01 on Other Chilli Varieties

CFPL01 was capable of infecting and causing disease symptoms in all tested chilli varieties (Fig. 2c). The DI% values ranged from 80%–100%. The highest DSI% value was observed in the Kheenoo Suan variety (61%), followed by Num (60%), Yuak (55%), red Chee Fah (39%) and red Jinda (21%). The finding indicated that CFPL01, known for its aggressiveness in causing chilli anthracnose, exhibited infection across a diverse range of hosts.

### Preliminary Screening of Antimicrobial Activity Against Phytopathogens

CF-SWUC02 exhibited potent antifungal activity at concentrations of 50% and 25%, resulting in complete inhibition of all the tested fungal isolates (Table 2). However, *Fusarium* sp. YHPB04-1 and YHPB05-1 were able to grow at the concentration of 25%, with MGI% values of 57 ± 6.77% and 59 ± 3.65%, respectively. Additionally, CF-SWUC02 exhibited antimicrobial activity against all phytobacteria tested, with the most pronounced effect observed against *Xcc*. The inhibition zone was 26 ± 0.49 mm. The inhibition zones observed against *A. tumefaciens*, *P. carotovora* and *R. solanacearum* were 9 ± 1.05 mm, 9 ± 1.30 mm and 7 ± 0.66 mm, respectively.

### Efficacy of CF-SWUC02 on Mycelial Growth and Conidial Germination of *C. truncatum* CFPL01

**Figure 3:**
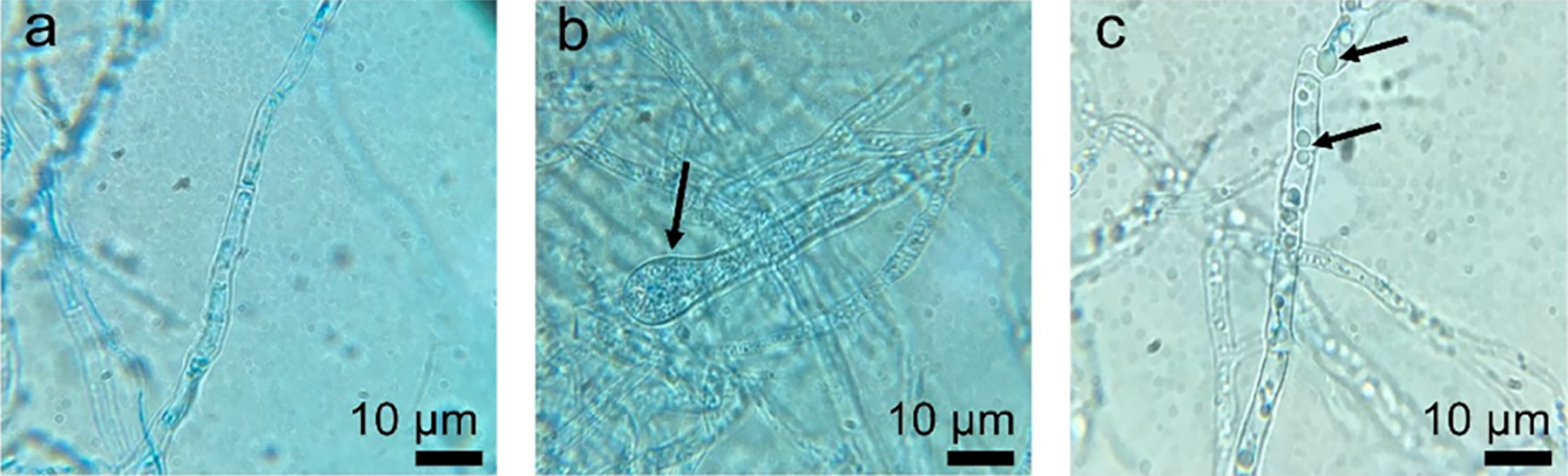
Hyphae morphology of *C. truncatum* CFPL01 mycelia incubated under different conditions. (a) PDB (control). Black arrows indicated (b) hyphal swelling and (c) vacuolation of *C*. *truncatum* CFPL01 hyphae when treated with 50% CF-SWUC02.

CF-SWUC02, at a minimum concentration of 12.5%, exhibited complete inhibition of fungal mycelial growth and conidial germination (Table 3). At a concentration as low as 1.56%, CF-SWUC02 demonstrated considerable inhibitory effects on fungal growth, achieving an MGI value reduction of 8%. CF-SWUC02 induced morphological abnormalities in the confronted mycelia, characterised by vacuolation and swelling, in contrast to the control treatment, where the mycelia were incubated with 0.05% v/v Tween 80 solution (Fig. 3). The conidial germination rate decreased significantly when incubated with CF-SWUC02 at or below 6.25% (Table 3).

### Antifungal Activity of the Extracts and Determination of MIC and MFC against *C. truncatum* CFPL01

At a concentration of 200 μg/mL, the dichloromethane extract (dCF) inhibited the mycelial growth of CFPL01 by 54 ± 3.55% on PDA (Table 4). The ethyl acetate extract (eCF) and *n*-hexane extract (hCF) also exhibited antifungal activity with MGI% values of 43 ± 3.35% and 16 ± 5.15%, respectively. The MIC values of dCF, eCF and fungicide mancozeb against CFPL01 were 62.5 μg/mL, 1,000 μg/mL and 31.25 μg/mL, respectively (Table 4). All MFC values were the same concentration as their respective MIC values. Mancozeb exhibited the highest activity, followed by dCF and eCF. However, even at a concentration of 1,000 μg/mL, hCF, benomyl and carbendazim did not completely inhibit fungal growth. These findings suggest that dCF exhibited the highest antifungal activity among the extracts, followed by eCF and hCF, indicating the presence of bioactive compounds with varying degrees of effectiveness.

### Effect of the Extracts on Reducing Anthracnose Severity in Yellow Chilli Leaves and Seedlings

**Figure 4:**
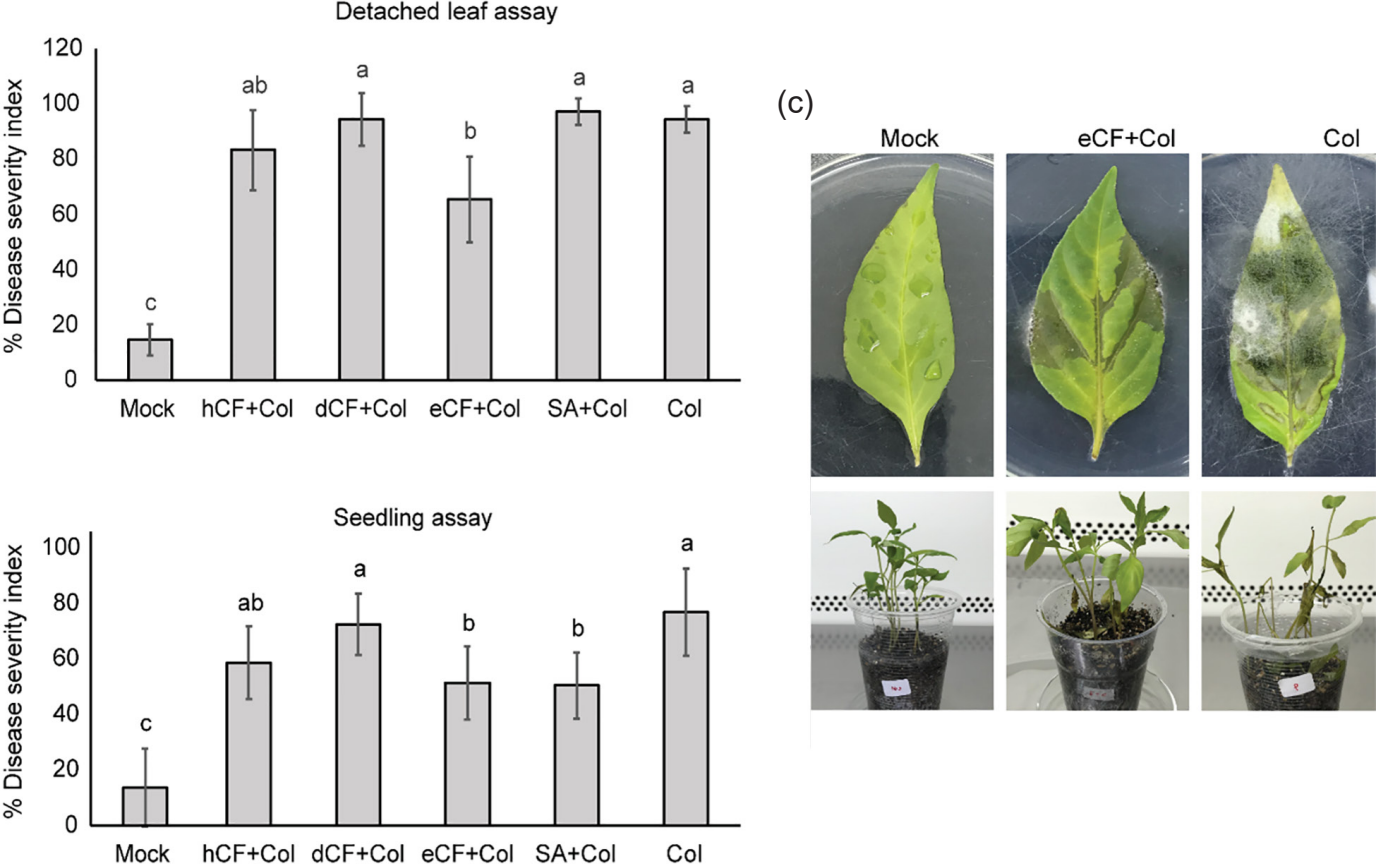
Effect of the extracts on anthracnose disease severity. (a) Percentage of disease severity index of detached leaf and (b) seedling assays. (c) Disease symptoms on detached leaves (upper) and seedlings (lower) that were either treated or not treated with eCF prior to the pathogen infection. The bars represent the means with standard deviation. Different letters indicate significant differences among the treatments within each assay based on the Mann-Whitney U test (*p* < 0.05). The abbreviations hCF, dCF, eCF, SA and Col represent *n*-hexane extract, dichloromethane extract, ethyl acetate extract, salicylic acid and the pathogen CFPL01, respectively.

In the detached leaf assay, only the eCF treatment demonstrated a 31% reduction in disease severity compared to the Col treatment, indicating a decrease in disease severity (Figs. 4a and 4c). No significant differences were observed when hCF, dCF or SA were applied. In contrast, the eCF-treated and SA-treated seedlings showed a 33% and 34% reduction in severity, respectively, compared to the Col treatment. The DSI% values of hCF and dCF were comparable to those of the Col treatment (Figs. 4b and 4c). These findings suggest that eCF has the potential to significantly reduce disease severity in both detached leaves and yellow chilli seedlings.

### Phenylalanine Ammonia-Lyase Activity

**Figure 5:**
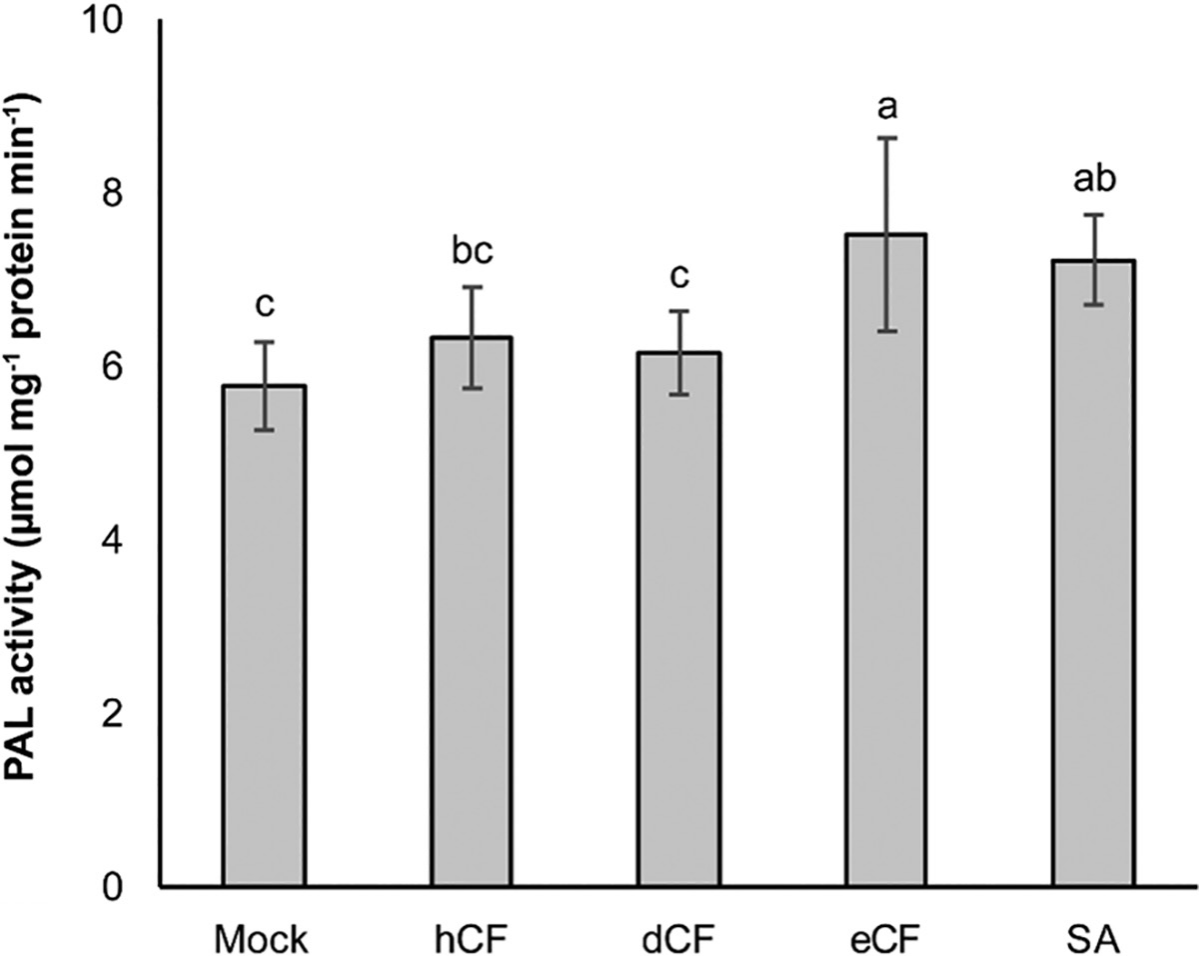
Phenylalanine ammonia-lyase (PAL) activity of the yellow chilli seedlings that were untreated (mock), treated with the CF-SWUC02 extracts, or treated with the elicitor SA. The bars represent the means with standard deviation. The letters indicate significant differences among the treatments within each assay based on the LSD test (*p* < 0.05). The abbreviations hCF, dCF, eCF and SA represent *n*-hexane extract, dichloromethane extract, ethyl acetate extract and salicylic acid, respectively.

After 48 h of treatment, only eCF induced PAL activity at a level comparable to that of the elicitor SA. This was significantly different from the mock treatment and treatments with other extracts. In contrast, both the hCF and dCF did not exhibit a significant difference in PAL activity when compared to the mock treatment, despite the fact that dCF exhibited the highest antifungal activity (Fig. 5).

## DISCUSSION

*Pseudomonas* spp. have been extensively studied for their efficacy in managing plant diseases and promoting plant growth. CF-SWUC02 derived from *P. aeruginosa* SWUC02 has demonstrated broad-spectrum antimicrobial activity, effectively inhibiting various fungi and bacteria commonly encountered in agriculture. Therefore, it is potentially suitable for agricultural use in plant disease control. The isolated fungi including *Alternaria*, *Fusarium*, *Cladosporium* and *Stemphylium* have previously been reported to be associated with diseases affecting chilli crops (Liu *et al*. 2022). Additionally, *A. tumefaciens, P. carotovora*, *R. solanacearum* and *Xanthomonas* spp. have been identified as some of the most important bacterial phytopathogens (Mansfield *et al*. 2012). Two isolates of *Fusarium* demonstrated high resistance to 25% CF-SWUC02. Notably, *Fusarium graminearum* can form biofilms offering protection against fungicides (Shay *et al*. 2022). Additionally, CF-SWUC02 exhibited the most effective antibacterial activity against *Xcc*. This observation can be attributed to the primary investigation conducted on *P. aeruginosa* SWUC02, which was initially isolated from lime plants and screened for its potential to suppress canker disease in lime plants (Sudyoung *et al*. 2020a).

Utilising both fungal morphology and molecular characteristics, especially ITS, *TUB2* and *GAPDH* regions, successfully identified *C. truncatum* CFPL01 and CFPL02. Their pathogenicity was confirmed through Koch’s postulate in yellow chilli fruits, leaves and seedlings. The capable of cross-infecting different chilli varieties suggests that CFPL01 is a virulent strain and could potentially lead to disease outbreaks. The most susceptible variety was the *C. frutescens* variety Kheenoo Suan, compared to *C. annuum* varieties. It is consistent with the study conducted by Montri *et al*. (2009). Among the tested fungicides, the dithiocarbamate fungicide mancozeb was the most effective, which acts as a sulfhydryl-enzyme inhibitor to disrupt biochemical processes within the fungal cells. Previous reports have identified and documented benzimidazole fungicide-resistant *Colletotrichum* species due to β-tubulin mutations in Thailand and other countries (Ridzuan *et al*. 2018; Poti *et al*. 2020). Our findings also demonstrated that the pathogen exhibited resistance to benzimidazole fungicides carbendazim and benomyl.

Cell-free cultures and bioactive compounds from *Pseudomonas* spp. have attracted attention owing to their ability to inhibit phytopathogens (Khan *et al*. 2022; Lahkar *et al*. 2018). Pseudomonads are also well-known producers of several compounds with antimicrobial and/or elicitor activities (Munhoz *et al*. 2017). Our study revealed that the bioactive compounds extracted from CF-SWUC02 using different solvents exhibited diverse activities. The dichloromethane extract (dCF) exhibited superior antifungal activity against CFPL01, outperforming carbendazim and benomyl. Additionally, eCF effectively induced PAL activity and disease resistance in yellow chilli seedlings, similar to the effects of SA. Previous reports have shown that the dichloromethane extracts derived from *P. aeruginosa* supernatant contain phenazine-1-carboxylic acid (PCA), phenazine-1-carboxamide (PCN), and OAC, which exhibit strong antibiotic activity (Simionato *et al*. 2017; Munhoz *et al*. 2017). Khan *et al*. (2022) reported the presence of quinolones, cascaroside B, 3,9-dimethoxypterocarpan and pyochelin in the ethyl acetate extract of *P. aeruginosa* Ld-08. PCN has also been reported to enhance the SAR response in tomato plants by inducing defense-related enzymes, including PAL, peroxidase and polyphenol oxidase (Munhoz *et al*. 2017). Endolysin and lokisin derived from *Pseudomonas* can trigger ISR in rice plants and increase disease resistance against the rice blast pathogen *Magnaporthe oryzae* (Omoboye *et al*. 2019). Fatima and Anjum (2017) reported that HMB extracted from *P. aeruginosa* PM12 culture using ethyl acetate exhibited the potential for ISR elicitation against Fusarium wilt in tomato plants. Our study observed that eCF was more effective than dCF in controlling anthracnose disease in seedlings. This suggests that in addition to directly inhibiting the fungus, triggering strong plant immunity to combat the pathogen is a crucial strategy. However, further research is necessary to identify the active compounds and to evaluate the efficacy of the extract under field conditions.

## CONCLUSION

In summary, our study highlights the significant potential of CF-SWUC02, derived from *P. aeruginosa* SWUC02, and its extracts as promising biocontrol agents against virulent *C. truncatum* CFPL01, caused anthracnose in several chilli varieties. CF-SWUC02 exhibited broad antimicrobial activity against a range of fungal and bacterial phytopathogens. The bioactive compounds present in dCF effectively inhibited the growth of CFPL01. Moreover, the bioactive compounds in eCF not only inhibited the growth of CFPL01, but also acted as a potent elicitor, inducing the plant’s defense mechanism. These findings suggest that these extracts have the potential to act as viable alternatives to conventional fungicides, offering effective management of anthracnose while supporting sustainable agricultural practices.

## Figures and Tables

**Table 1:** Disease severity scores.

Assay	Severity scores	Description
Fruits	0	Healthy (without symptoms)
	1	Fruit showing small lesions up to 0.2 cm^2^
	2	Fruit showing lesion area > 0.2 cm^2^–1 cm^2^
	3	Fruit showing lesion area > 1 cm^2^–2 cm^2^
	4	Fruit showing lesion area > 2 cm^2^
Detached leaf	0	Healthy
	1	Large water-soaked lesions (possible acervuli)
	2	Lesions covering 20%–40% of leaf, up to 5% with acervuli
	3	Lesions covering > 40% of leaf, up to 25% with acervuli
	4	Lesions covering > 70% of leaf, > 25% with abundant acervuli
Seedling	0	Healthy
	1	25% leaves with yellowing/necrosis/defoliation
	2	25%–50% affected leaves
	3	50%–75% affected leaves or level 2 with stem lesions
	4	> 75% affected leaves or level 3 with stem lesions
	5	Level 4 with stem lesions and all unhealthy leaves
	6	Dead seedlings

**Table 2:** Antifungal and antibacterial activities of CF-SWUC02 against a broad range of phytopathogens.

Tested microorganisms	Mycelial growth inhibition (MGI%)		Inhibition zone (mm)

	50% CF-SWUC02	25% CF-SWUC02	
*Cladosporium* sp. YHPM01-1	100 ± 0.00	100 ± 0.00^a^	–
*Alternaria* sp. YHPM02-2	100 ± 0.00	100 ± 0.00^a^	–
*Alternaria* sp. YHPM03-1	100 ± 0.00	100 ± 0.00^a^	–
*Fusarium* sp. YHPB04-1	100 ± 0.00	57 ± 6.77^b^	–
*Fusarium* sp. YHPB05-1	100 ± 0.00	59 ± 3.65^b^	–
*Stemphylium* sp. YHPM06	100 ± 0.00	100 ± 0.00^a^	–
*Colletotrichum truncatum* CFPL01	100 ± 0.00	97 ± 3.76^a^	–
*C. truncatum* CFPL02	100 ± 0.00	100 ± 0.00^a^	–
*A. tumefaciens*	–	–	9 ± 1.05^B^
*P. carotovora*	–	–	9 ± 1.30^B^
*R. solanacearum*	–	–	7 ± 0.66^C^
*X. citri* subsp. *citri*	–	–	26 ± 0.49^A^

*Notes:* The means and standard deviations are shown. Different lowercase letters in the column indicate significant differences among the microorganisms based on the Mann-Whitney U test (*p* < 0.05), whereas different uppercase letters represent significant differences based on the LSD test (*p* < 0.05).

**Table 3:** Inhibitory effects of CF-SWUC02 on mycelial growth and conidial germination of *C. truncatum* CFPL01.

CF-SWUC02 (%)	Mycelial growth inhibition (MGI%)	Conidial germination inhibition (CGI%)
50.00	100 ± 0.00^a^	100 ± 0.00^A^
25.00	100 ± 0.00^a^	100 ± 0.00^A^
12.50	100 ± 0.00^a^	100 ± 0.00^A^
6.25	50 ± 5.78^b^	96 ± 0.58^B^
3.13	25 ± 3.96^c^	91 ± 4.58^C^
1.56	8 ± 3.54^d^	92 ± 2.52^C^

*Notes:* The means and standard deviations are shown. Different lowercase letters in the column indicate significant differences in MGI% at specific CF-SWUC02 concentration, while different uppercase letters represent significant differences in CGI% at the corresponding CF-SWUC02 concentrations, as determined by the Mann-Whitney U test (*p* < 0.05).

**Table 4:** MGI%, MIC and MFC values of the extracts and fungicides against *C. truncatum* CFPL01.

Extract/Fungicide	MGI% at 200 μg/mL extracts	MIC (μg/mL)	MFC (μg/mL)
hCF	16 ± 5.15%	> 1,000	> 1,000
dCF	54 ± 3.55%	62.5	62.5
eCF	43 ± 3.35%	1,000	1,000
Benomyl 50 WP	–	> 1,000	> 1,000
Carbendazim 50 WP	–	> 1,000	> 1,000
Mancozeb 80 WP	–	31.25	31.25

*Notes:* The abbreviations hCF, dCF and eCF represent *n*-hexane, dichloromethane and ethyl acetate extracts, respectively.
